# Unravelling Cu_6_Sn_5_ precipitate coarsening mechanisms in SAC solders under thermomechanical cycling

**DOI:** 10.1038/s41598-025-21633-y

**Published:** 2025-10-28

**Authors:** Charlotte Cui, Sebastian Krauß, Hooman Hosseinkhannazer, Julien Magnien, Olena Vertsanova, Michael Reisinger, Peter Imrich, Walter Hartner, Roland Brunner

**Affiliations:** 1 Department Microelectronics, Materials Center Leoben Forschung GmbH, Vordernberger Straße 12, Leoben, 8700 Austria; 2 Carl Zeiss Microscopy GmbH, Carl-Zeiss-Straße 22, 73447 Oberkochen, Germany; 3 Norcada Inc, 4548 99 Street NW, Edmonton, AB T6E 5H5 Canada; 4 Kompetenzzentrum für Automobil- und Industrieelektronik GmbH, Europastraße 8, Villach, 9524 Austria; 5 Infineon Technologies AG, Wernerwerkstraße 2, 93049 Regensburg, Germany

**Keywords:** Electronic devices, Imaging techniques

## Abstract

Thermo-mechanical cycling of microelectronic devices creates complex stress-states in Sn–Ag–Cu (SAC) solder balls, leading to Cu₆Sn₅-precipitate coarsening. Two key mechanisms — strain-induced coarsening and Ostwald ripening — are examined separately. Strain-induced coarsening, studied via plastic shear deformation, is more significant in dynamically recrystallised high-strain regions than in lower-strain shear band regions. Ostwald ripening is investigated via in-situ FESEM, and its interplay with strain-enhanced coarsening is analysed in thermo-mechanically cycled solders with varying Bi-contents. Results show that Bi, solved in the β-Sn matrix, delays dynamic recrystallisation and reduces both strain-enhanced coarsening and Ostwald ripening of Cu₆Sn₅. Nonetheless, Cu_6_Sn_5_-precipitates are 1.5–3 times larger in recrystallised high-strain regions than in single-crystalline lower-strain regions regardless of Bi-content, due to strain-enhanced coarsening during thermo-mechanical cycling. The findings indicate that mechanical strain plays a dominant role in precipitate growth, suggesting that strain-enhanced Cu_6_Sn_5_ coarsening, and thusly decreased precipitate strengthening effects, correlate with increased thermo-mechanical fatigue.

**Supplementary Information:**

The online version contains supplementary material available at 10.1038/s41598-025-21633-y.

## Introduction

Structural and functional metal alloys may experience high homologous temperatures during service^1–4^, examples comprising components in high-temperature aerospace^1,2^, power semiconductor^3^ or automotive^1,5^ applications. The elevated temperatures can be further superimposed with mechanical strain, ultimately increasing the load on the material or component. Thus, operational temperature ranges strongly depend on the nature of the material, i.e. its mechanical properties and associated microstructural features at the given temperature^4,6–8^. Naturally, for low-melting metals, associated mechanisms will be triggered in less harsh conditions than for high-melting ones. Ni-base superalloys, for instance, undergo creep above 700 °C^4,7^. Conversely, low-melting Sn-based solder alloys, utilised to interconnect non-melting or heat-sensitive materials e.g. in microelectronic devices^9–12^, have eutectic temperatures below 250°C^9,13–15^ and consequently experience creep well below 100 °C ^16–18^. Nonetheless, the fundamental mechanisms governing microstructural changes, e.g. precipitate coarsening, may be similar for high- and low-melting metals under homologous load^19,20^. Moreover, scrutinising strain-induced precipitate coarsening and its interplay with thermally activated coarsening is essential to gain in-depth understanding about underlying mechanisms and to further engineer failure resistant material designs^4,6–8^. In order to fully comprehend the microstructural evolution during thermo-mechanical load, it is crucial to unravel the effects of thermal and mechanical loads on the microstructure. Hence, by studying these loads separately, the microstructural degradation mechanisms that each one induces, can be clearly identified and quantified. Consequently, by separately studying thermal and mechanical effects on microstructural degradation, clearer insights into failure processes and root causes can be gained, and fatigue can be modelled more accurately. Due to similarities in the underlying precipitate coarsening mechanisms like Ostwald ripening during thermal ageing^19,20^ and strain-induced coarsening caused by dislocation pipe diffusion^6,21^, the systematic study of low-melting, low-strength metal alloys could also provide highly valuable insights about precipitate coarsening in high-melting, high-strength ones. Furthermore, in-situ studies could open up various possibilities. Indeed, directly observing the microstructural changes caused by such mechanisms during in-situ studies is complex, in particular for high-melting metals due to the corresponding high temperatures involved, demanding highly sophisticated experimental setups. For low-melting metals, in-situ investigations can be performed in experimentally more accessible temperature ranges^22–26^.

Microelectronic devices often consist of multi-material, multi-layer stacks and undergo several temperature changes during their lifetime due to changes in the environment as well as due to resistive heating^13,27–33^. Because of the inherently different thermal expansion coefficients of the materials involved, thermal misfit stresses develop within the device during such temperature changes. In order to interconnect individual building blocks, i.e. chips and printed circuit boards (PCBs) into a functioning electronic device, Sn – Ag – Cu (SAC-) solders are frequently utilised due to their non-toxicity^34–36^ and processability^3,9,28^. During temperature changes however, these solder balls may absorb large proportions of plastic strains caused by thermal expansion misfits, because of their comparatively low yield strengths^10,37^. In order to elevate the solder ball’s yield strength, Bi can be additionally alloyed with contents below its solubility limit in β-Sn of ~ 2.5 wt% ^10,37,38^. Thus, Bi acts as a solid-solution strengthener in the β-Sn matrix for such contents^10,39^. Due to the low eutectic temperature of SAC305-solder balls of 217 °C ^13,14^, which is even lower for Bi-containing alloys^9,10^, they may additionally experience creep during high-temperature loading periods. Hence, complex load cases evolve in SAC-solder balls during temperature changes. In order to study the fatigue behaviour of SAC-solder balls during such loads, thermo-mechanical cycling experiments can be conducted^3,13,27,28,33^. Herein, entire microelectronic devices are subjected to periodic temperature cycles, whereas the mechanical strain stems only from the inherent thermal expansion misfits which evolve during these cycles^14,27,28,31,40,41^. Within the solder balls, misfit stresses during thermo-mechanical cycling are distributed inhomogeneously, but are generally highest near the chip- and PCB-interfaces^27,31,33,39^.

The microstructural makeup of a SAC-solder ball mainly consists of a β-Sn matrix, in which intermetallic phases, namely Ag_3_Sn and Cu_6_Sn_5,_ are embedded^9,10,13,28,42^. In the as-reflowed condition, the Cu_6_Sn_5_-precipitates form a fine ternary eutectic, together with β-Sn and Ag_3_Sn, in the interdendritic spaces of β-Sn^9,10,13,28,42^ which solidifies in a single-crystalline or few-grained structure^27,30,40,43,44^. During thermal ageing, these precipitates coarsen due to Ostwald ripening^45^. Moreover, they are also observed to coarsen during thermo-mechanical fatigue^27,32^. The coarsening of these precipitates may decrease the yield strength of the solder ball^19,20,46,47^, which may have adverse effects on its mechanical integrity. This may have consequential impacts on the thermo-mechanical fatigue behaviour of these solder balls, wherein they undergo periodic mechanical strain from thermal expansion misfits in the multi-layer device^3,27,41,48^. Any softening of the SAC-solder balls may accelerate fatigue crack initiation and propagation, which will cause failure of the microelectronic device^27,45^. In addition to Ostwald ripening, Cu_6_Sn_5_-precipitates may grow due to strain-induced coarsening^21,46^ caused by thermal misfit stresses during thermo-mechanical cycling^3,13,27,33^, which may further accelerate the microstructural degradation of SAC-solder balls. Interplay and overlay of these various mechanisms, which facilitate Cu_6_Sn_5_-precipitate coarsening during thermo-mechanical cycling, may muddy their effects when they are examined simultaneously.

Therefore, the aim of this study is the disaggregated but correlative analysis of strain-induced coarsening and Ostwald ripening as well as the evaluation of the interplay between both mechanisms during thermo-mechanical cycling and the influence of solid-solution strengthening by Bi. For the study of strain-induced coarsening shear deformation of as-reflowed solder balls is performed at room temperature, as they mostly experience shear stresses during thermo-mechanical cycling^48^. Ostwald ripening, on the other hand, is studied via in-situ ageing utilising field emission scanning electron microscopy (FESEM) in combination with a microelectromechanical system (MEMS) for in-situ heating. The in-situ experiments yield invaluable information about microstructural evolution under controlled loading conditions, which is especially crucial for the complex load case of the small solder balls during thermo-mechanical cycling^49–51^. Finally, the Cu_6_Sn_5_-precipitates embedded in the SAC microstructure are statistically analysed after thermo-mechanical cycling. Based on the quantification of Cu_6_Sn_5_-precipitate sizes, we systematically show that the effect of strain-induced coarsening increases with increasing degrees of plastic deformation of the as-reflowed solder balls, i.e. increasing shear forces. This is also true for deformation gradients within individual solder balls, wherein highly deformed, dynamically recrystallised areas exhibit larger Cu_6_Sn_5_-precipitates than less-deformed shear band regions. After thermo-mechanical cycling, Cu_6_Sn_5_-precipitates in dynamically recrystallised high-strain areas of the solder balls of all Bi-contents are found to be ~ 150–300% larger than in the single-crystalline lower-strain areas, while overall precipitate sizes decrease with increasing Bi-content with a concurrent decrease in dynamic recrystallisation and fatigue cracking. Ultimately, this study reveals that the growth of larger Cu_6_Sn_5_-precipitates during in-situ ageing is at the cost of shrinkage and consumption of smaller ones, a typical evidence for Ostwald ripening^19,20,45^. Based on the presented results, it is concluded that strain-induced coarsening significantly impacts the growth of Cu_6_Sn_5_-precipitates during thermo-mechanical cycling, which may impair their precipitation strengthening effects and accelerate fatigue. It is shown, that the addition of Bi reduces Cu_6_Sn_5_-precipitate coarsening, as well as dynamic recrystallisation and fatigue.

## Results

### Microstructural changes in as-reflowed solder balls upon plastic shear deformation

**Fig. 1:**
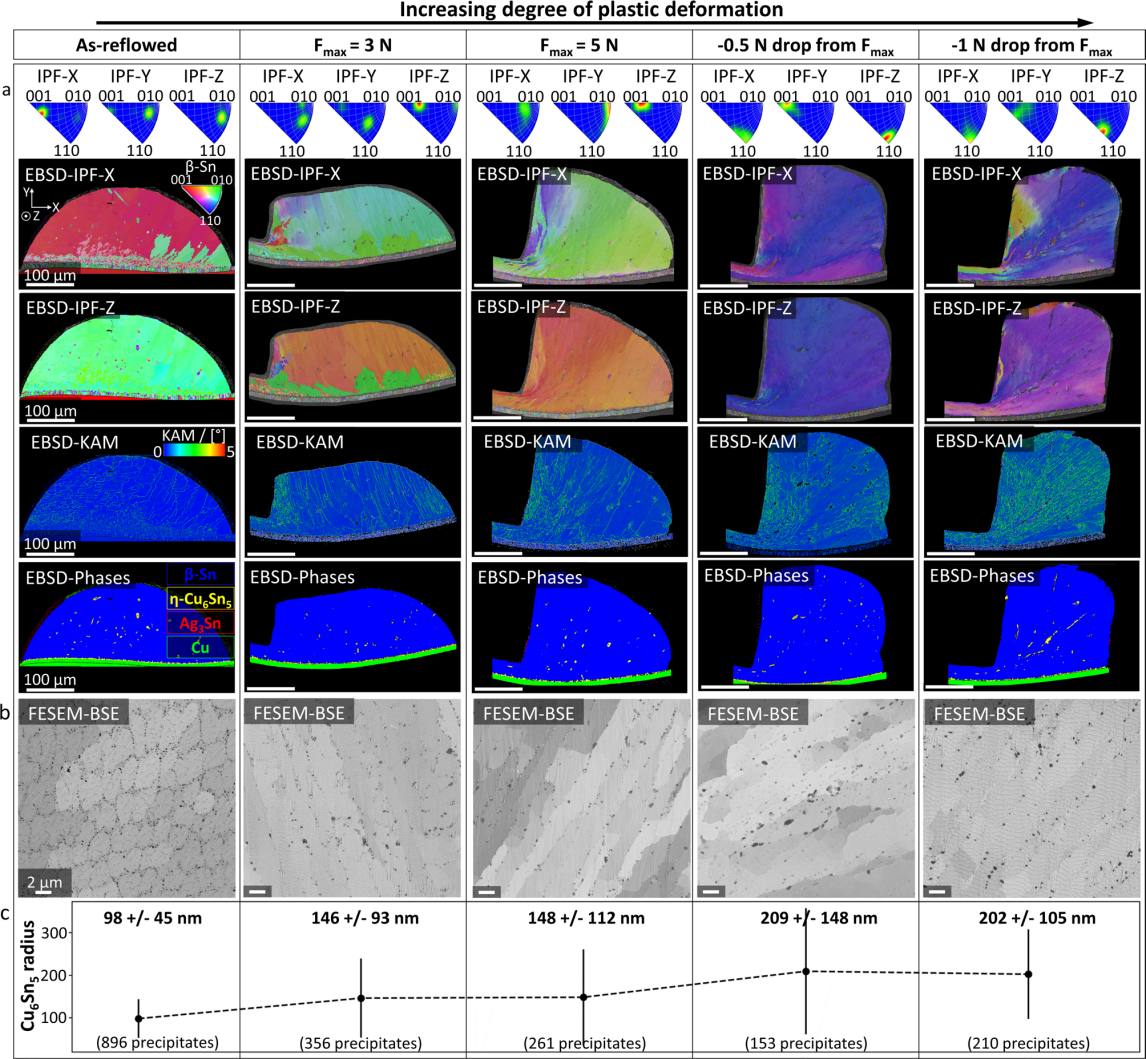
**Cu**_**6**_**Sn**_**5**_**-precipitate coarsening due to plastic shear deformation at room temperature.** Microstructural evolution in as-reflowed solder balls after plastic shear deformation at room temperature. From left to right: Increasing degree of deformation. **a** EBSD mappings of solder balls after various degrees of plastic shear deformation. IPFs, IPF mappings for X and Z, as well as KAM mappings and phase mappings are shown. In the phase mappings, β-Sn is coloured blue, Cu_6_Sn_5_ in yellow, Ag_3_Sn in red and Cu in green. Increasing misorientation is discernible with increasing deformation in the IPF mappings, as well as increasing misorientation in the KAM mappings. Moreover, misorientation gradients are also visible within the KAM mappings of each ball. Scalebars of 100 μm are valid for all images. **b** FESEM-BSE details of the solder balls are shown in **a**. Sn, Cu_6_Sn_5_ and Ag_3_Sn are distinguishable due to mass-contrast. Due to the large proportion of Cu, Cu_6_Sn_5_ illustrates the darkest phase in the image. Scalebar of 2 μm is valid for all images. **c** Equivalent radii of Cu_6_Sn_5_-precipitates obtained from the micrographs, shown in **b, **are illustrated. Further, mean values, standard deviations, and precipitate counts are provided.

The effects of mechanical strain on Cu_6_Sn_5_-precipitate coarsening during thermo-mechanical fatigue are separately studied by performing plastic shear deformation of as-reflowed SAC-solder balls at room temperature. Exemplary cross-sectional micrographs of the solder balls are shown in Fig. 1. Four various degrees of deformation are investigated, defined by the maximum shear force (F_max_) that is applied during the respective experiment. In Fig. 1, the degree of deformation increases from left to right. One undeformed as-reflowed solder ball is also included as reference. Further details about the shear deformation experiments are given in **Methods** and **Supplementary Fig. 1**. Figure 1a illustrates an overview of the electron backscatter diffraction (EBSD) inverse pole figure (IPF) and kernel average misorientation (KAM) mappings for each degree of deformation. In the as-reflowed condition, the investigated solder balls consist primarily of only one large β-Sn grain. In the as-reflowed and 3 N sample, some smaller β-Sn grains are present, as discernible from the IPF-X and IPF-Z mappings, respectively, as well as the phase mappings in Fig. 1a. However, these smaller grains only exhibit misorientation to the primary grain in one direction, as shown in the corresponding IPF-Z and IPF-X mappings. Morphologically, these grains appear consistent with the interlaced as-reflowed structure reported in^27^.

The EBSD-IPF and EBSD-KAM mappings in Fig. 1a further illustrate that the misorientation within the balls increases with increasing shear deformation. Moreover, various zones of deformation are discernible within the β-Sn matrix of the solder balls. Recrystallised high-deformation zones are located around the contact zone of the shear-tool, where the misorientations are the highest, as well as ordered structures further away from the contact zones, where the highest misorientation values are localised in shear bands. Figure 1b shows FESEM backscatter electron (BSE) details of these shear band zones, as well as the as-reflowed structure as reference. By utilising FESEM-BSE signals, individual shear bands are discernible due to channelling contrast, whereas the β-Sn matrix, Cu_6_Sn_5_- and Ag_3_Sn-precipitates are distinguishable due to mass contrast. Hence, Cu_6_Sn_5_-precipitates appear as the darkest phase. Based on this mass contrast, the Cu_6_Sn_5_-precipitates are segmented by their grey-values and their equivalent radii are calculated. Mean values, as well as standard deviations of these calculations are presented in Fig. 1c. Overall, an increase of the Cu_6_Sn_5_-precipitate size is observed with increasing degree of deformation. A stagnation of coarsening is observed between the two highest deformation degrees, i.e. −0,5 and − 1 N drops from F_max_, as discernible in Fig. 1c. The fact that this observation is not strictly monotonic might be attributed to differences in deformation mechanism, as suggested by differences in the KAM mappings in Fig. 1a. Moreover, the force – time-curves in **Supplementary Fig. 1c** depict a higher F_max_ for the − 0.5 N sample than for the − 1 N sample, suggesting variation in material response upon deformation. This may be caused by slight differences in the shear tool’s contact position or the as-reflowed geometries of the solder balls. However, both mean values lie within the standard deviation of the other, respectively, suggesting that strain-induced coarsening of Cu_6_Sn_5_-precipitates at room temperature also might saturate at large degrees of deformation.Nonetheless, an overall trend of increasing Cu_6_Sn_5_-precipitate size with increasing deformation is observed in Fig. 1c. Details about EBSD mapping and FESEM-BSE imaging are given in **Methods**. The threshold-based segmentation of Cu_6_Sn_5_-precipitates in FESEM-BSE micrographs, as well as the quantification and calculation of their equivalent radii are described in **Methods** and **Supplementary Note 1**.

### **Deformation gradient and strain-induced Cu**_**6**_**Sn**_**5**_**-coarsening at room temperature**

**Fig. 2:**
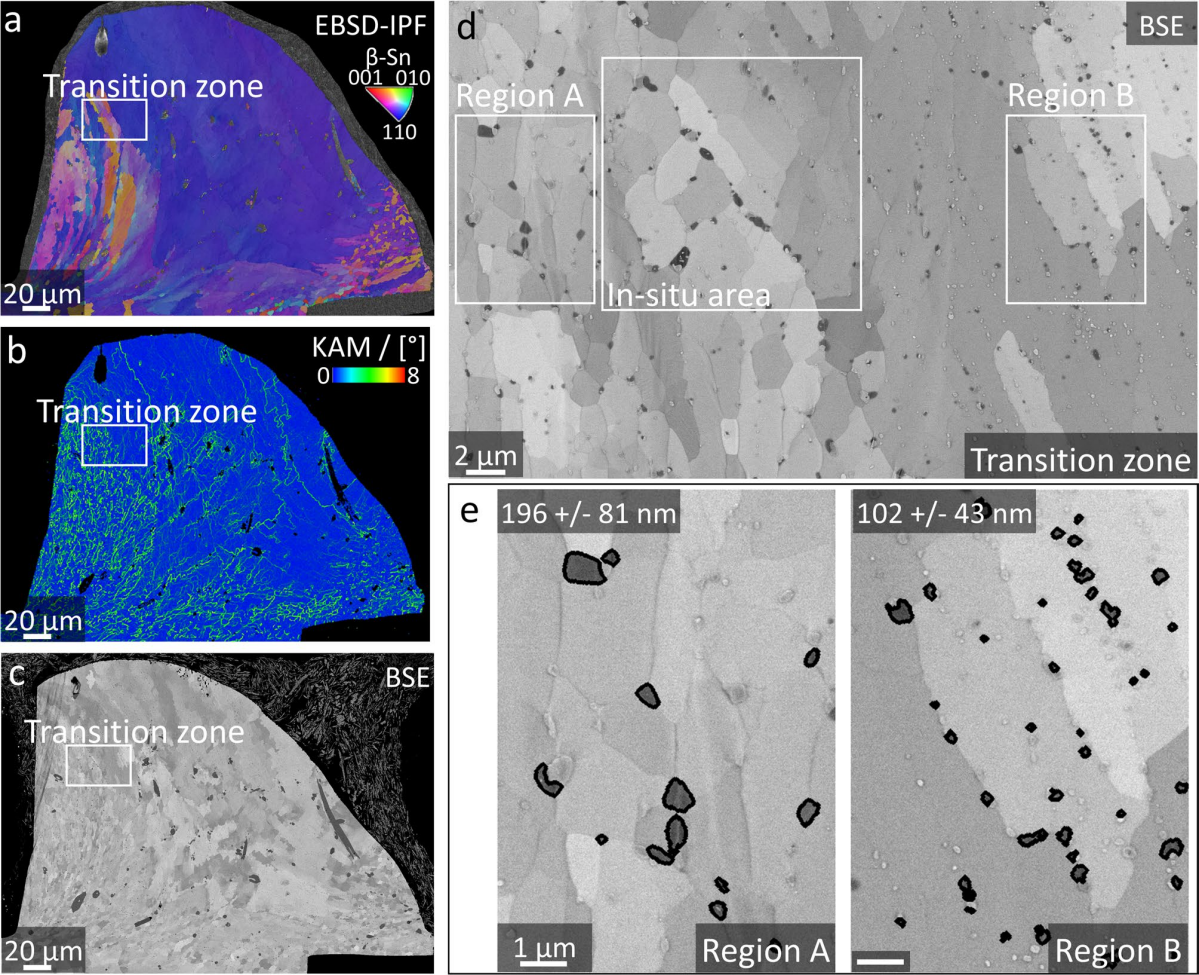
**Deformation gradient dependent Cu**_**6**_**Sn**_**5**_**-precipitate coarsening at room temperature.** Cu_6_Sn_5_-precipitate coarsening from plastic deformation at room temperature, as well as during in-situ FESEM ageing. **a** Overview EBSD-IPF and **b** EBSD-KAM mappings of the shear-deformed solder ball. The high- to low-deformation transition zone is marked by white rectangles in both mappings. **c** Overview FESEM-BSE micrograph of the shear-deformed solder ball. The transition zone is marked by a white rectangle. **d** FESEM-BSE micrograph of the transition zone. Regions A and B, marked with white rectangles, represent areas in the low- and high-deformation zones, respectively. Moreover, the in-situ area for in-situ FESEM ageing is also marked. **e** Image analysis of strain-induced Cu_6_Sn_5_-precipitate coarsening for region of interest A and B. Segmented Cu_6_Sn_5_-contours are overlaid onto the corresponding FESEM-BSE micrographs. Mean equivalent Cu_6_Sn_5_-precipitate radii and standard deviations are printed at the upper edge of each image. Scalebar of 1 μm is valid for both images.

Deformation gradients are present over the individual cross-sections of the plastically sheared samples. An exemplary sample that is deformed with a maximum shear force of 5 N is depicted in Fig. 2, in which various deformation zones are distinctly visible. The EBSD-IPF and -KAM mappings in Fig. 2a show the large initial grain in blue and a recrystallised, colourful area in the vicinity of the shear-tool contact zone. These two zones are also distinguishable in the EBSD-KAM mapping in Fig. 2b and the FESEM-BSE overview micrograph in Fig. 2c due to channelling contrast. The differences in grain structure imply that dynamic recrystallisation occurs in the high-strain area, whereas shear bands are formed in the lower-strain areas. A larger magnified FESEM-BSE micrograph of the transition zone between these two areas, marked with white rectangles in Fig. 2a, b and **c**, is depicted in Fig. 2d. Here, regions A and B, which represent a dynamically recrystallised area and a shear band area, are marked. Detailed FESEM-BSE micrographs of regions A and B are illustrated in Fig. 2e and the mean equivalent precipitate radii and standard deviations of Cu_6_Sn_5_-precipitates in regions A and B are printed in the top left corners. These equivalent radii are calculated based on binary threshold-based segmentation, the contours of which are overlaid onto the FESEM-BSE micrographs in black. The threshold-based segmentation of Cu_6_Sn_5_-precipitates in FESEM-BSE micrographs, as well as the quantitative calculation of their equivalent radii are described in **Methods** and **Supplementary Note 1**. The mean precipitate radius is 196 nm in the dynamically recrystallised region A, whereas in the shear band region of interest B it is only 102 nm.. Thus, strain-induced Cu_6_Sn_5_-precipitate coarsening is more pronounced in region of interest A than in B.

### In-situ ageing of the plastically deformed SAC-solder ball

**Fig. 3:**
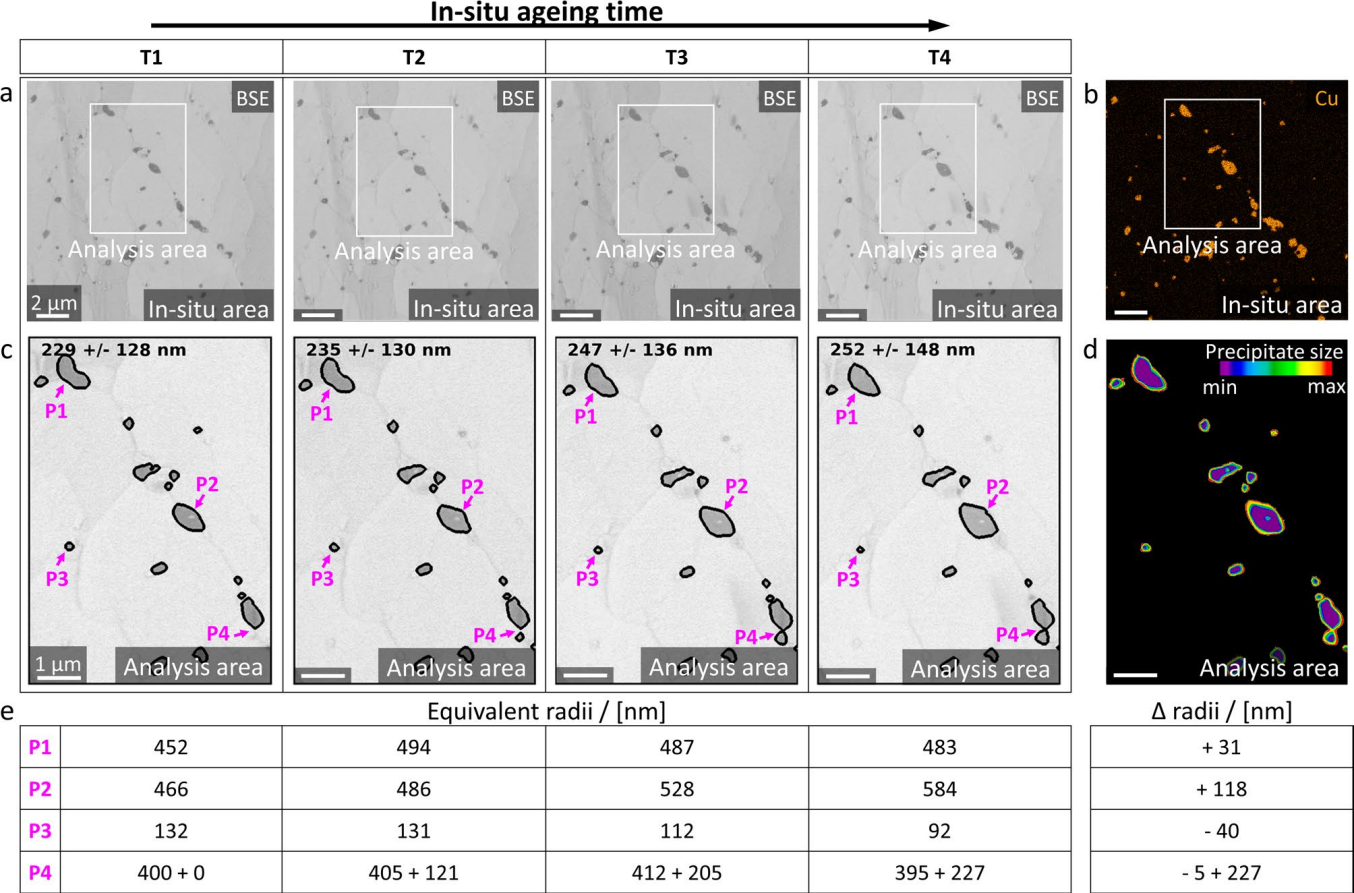
**In-situ FESEM ageing and Ostwald ripening of Cu**_**6**_**Sn**_**5**_**-precipitates.** In-situ Ostwald ripening of Cu_6_Sn_5_-precipitates in the in-situ area marked in Fig. 2d. **a** FESEM-BSE micrographs of the same area during the in-situ ageing experiment for timesteps T1 – T4, see **Supplementary Fig. 2**. Scalebar of 2 μm is valid for all images. **b** EDX-Cu mapping of **a** after in-situ FESEM ageing. The Cu signal is illustrated in orange. Scalebar of 2 μm is valid. **c** Cu_6_Sn_5_-precipitate growth in the analysis area from **a**. The contours of Cu_6_Sn_5_-precipitates are overlaid onto the FESEM-BSE micrographs in black and their mean equivalent precipitate radii, as well as standard deviations, are printed at the top of the image. Scalebar of 1 μm is valid for all images. Four precipitates (P1 – P4) are marked with pink arrows. **d** Visualisation of Cu_6_Sn_5_-precipitate changes from T1 to T4. The heatmap depicts precipitate sizes during in-situ ageing, wherein the respectively smallest size is shown in purple and the largest size in red. Hence, precipitate growth over time is shaded from purple to red and precipitate shrinkage vice versa. **e** Equivalent radii of P1 – P4 for each timestep, as well as their overall difference (Δ) between T4 and T1. P4 represents at the beginning a single precipitate but will eventually merge with its adjacent precipitate at the bottom. The equivalent radii for both are provided separately.

For the investigation of Cu_6_Sn_5_-precipitate coarsening during the high-temperature periods of thermo-mechanical cycling, in-situ FESEM ageing between 150 and 175 °C is performed on one representative shear-deformed sample for a total of 110 minutes. Figure 3 shows FESEM-BSE micrographs for four in-situ ageing timesteps depicting the microstructural changes in the in-situ area from Fig. 2d. Details about the in-situ setup, as well as the temperature profile are provided in **Methods**, **Supplementary Note 2** and **Supplementary Fig. 2**. The evolution of the in-situ area, marked in Fig. 2d, during the ageing experiment is illustrated in Fig. 3a. The Cu signal from electron dispersive X-ray spectroscopy (EDX) mapping of the in-situ area after in-situ FESEM ageing is exhibited in Fig. 3b. Details about EDX mapping are provided in **Methods**. Figure 3c depicts a detail from Fig. 3a, wherein morphological changes of the Cu_6_Sn_5_-precipitates are analysed. The mean equivalent precipitate radii and standard deviations are printed on top of each detail in Fig. 3c. Overall, precipitate coarsening is observed with increasing ageing time. For a more detailed description of the various ageing phenomena, four precipitates indicated by P1 to P4 and marked with pink arrows, are considered separately in each timestep. The overall change of the Cu_6_Sn_5_-precipitates over in-situ ageing time is illustrated in Fig. 3d as a heatmap, wherein the smallest precipitate areas during in-situ ageing are shown in purple and the largest ones in red. Hence, changes in the precipitate morphologies are visualised. Details about the heatmap calculation are given in **Methods**. Finally, the equivalent radii for each of the precipitates P1 – P4 at each timestep are provided in Fig. 3e. Among precipitates P1 – P4, various ageing phenomena are observed. P1, while maintaining a more or less constant size, reduces its curvature significantly during ageing. Additionally, various phenomena of Ostwald ripening according to^19,20^ occur. The initially already larger P2 continues to grow up to 125% of its initial size, increasing its radius by 118 nm, as shown in Fig. 3e, whereas the smaller P3 shrinks by 30%, reducing its radius by 40 nm. P4, initially a single precipitate, will eventually merge with its fast-growing adjacent neighbour at the bottom. EBSD-IPF and -KAM mappings of the in-situ area before and after ageing are provided in **Supplementary Fig. 3**.

#### Interplay between Bi-content, recrystallisation, strain-enhanced coarsening and Ostwald ripening of Cu_6_Sn_5_-precipitates during thermo-mechanical cycling

**Fig. 4:**
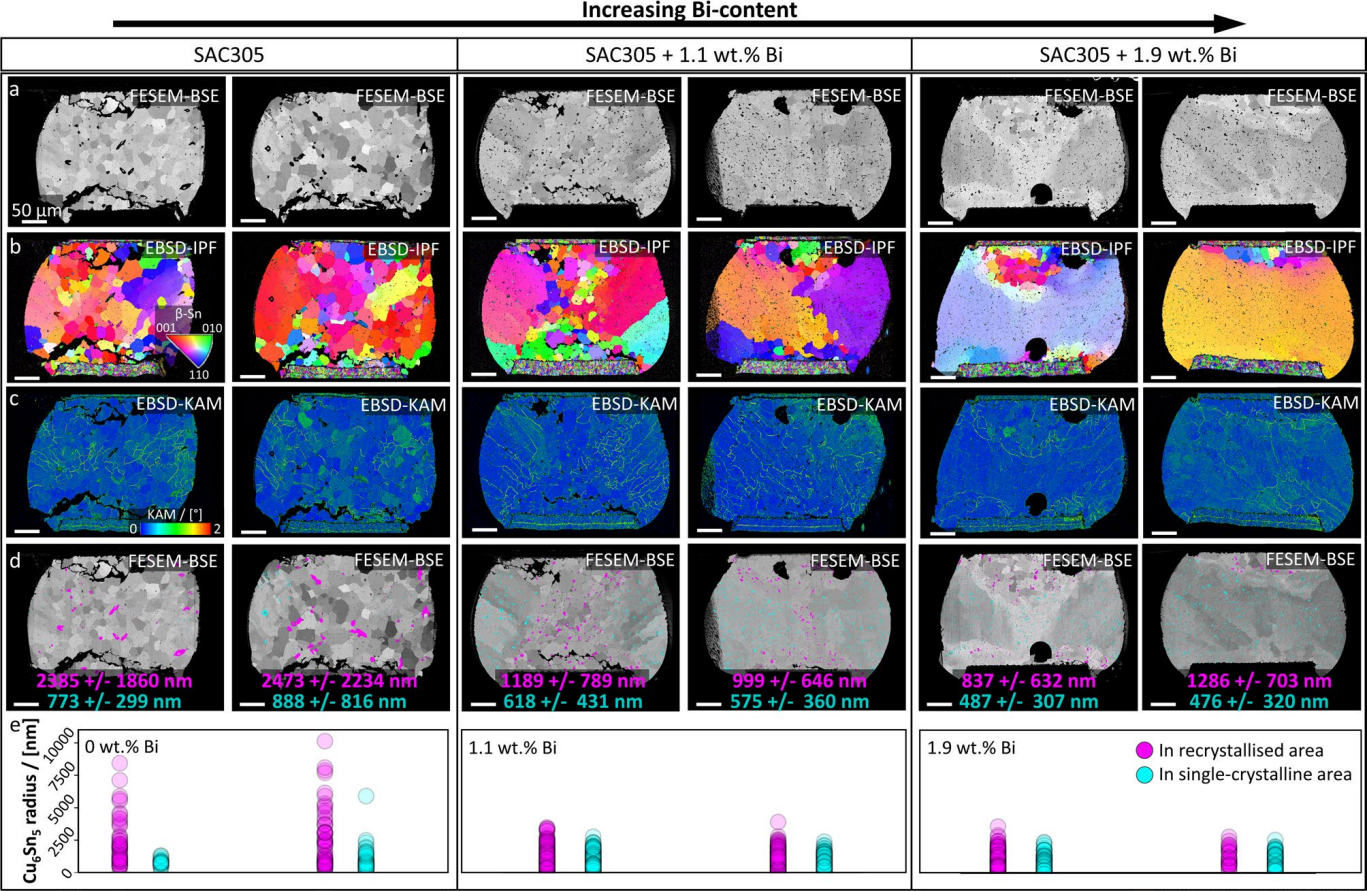
**Cu**_**6**_**Sn**_**5**_**-precipitate coarsening in high-strain areas of thermo-mechanically cycled SAC solder balls.** FESEM overview micrographs of representative thermo-mechanically SAC305, SAC305 + 1.1 wt% Bi and SAC305 + 1.9 wt% Bi solder balls. **a** FESEM-BSE micrographs. Recrystallised β-Sn grains are visible due to channelling contrast. Moreover, Cu_6_Sn_5_-precipitates appear darker due to mass contrast. Cu_6_Sn_5_-precipitates are enlarged in recrystallised areas. **b** EBSD-IPF mappings of the solder balls from **a**. Recrystallised areas from thermo-mechanical fatigue are shown. **c** Corresponding KAM mappings of the respective balls. Localised high-misorientation areas are visible in the not yet recrystallised areas. Scalebar of 50 μm is valid for all images. **d** Cu_6_Sn_5_-precipitates in recrystallised areas are coloured magenta, those in single-crystalline areas in cyan and overlaid onto the respective FESEM-BSE micrographs from **a**. Mean values, as well as standard deviations for Cu_6_Sn_5_-precipitates are evaluated separately for high-strain, recrystallised areas, printed in magenta, and for low-strain, single-crystalline areas, printed in cyan. For all depicted solder balls, the mean Cu_6_Sn_5_-precipitate sizes are larger in recrystallised areas compared to single-crystalline areas. **e** Cu_6_Sn_5_-precipitate size distributions in the micrographs in **a – d**. Cu_6_Sn_5_-precipitate radii are plotted in pink for ones situated in recrystallised areas and in cyan for ones in single-crystalline areas. Overall, precipitate sizes decrease with increasing Bi-content.

The interplay of strain-induced coarsening and Ostwald ripening of Cu_6_Sn_5_-precipitates during thermo-mechanical cycling is analysed in cross-sectional micrographs of SAC-solder balls. In addition to SAC305, also SAC305 + 1.1 wt% Bi as well as SAC305 + 1.9 wt% Bi solder balls are investigated in order to correlate Cu_6_Sn_5_-precipitate coarsening with increasing yield strengths of the β-Sn matrix, facilitated by the solid-solution strengthening effects of Bi. Figure 4a, b and c show FESEM-BSE overview micrographs, EBSD-IPF, as well as EBSD-KAM mappings of representative solder balls after thermo-mechanical fatigue, two for each Bi-content, respectively. Details about thermo-mechanical cycling are provided in **Methods**. Dynamically recrystallised areas are discernible in the FESEM-BSE micrographs, as well as in the EBSD-IPF mappings, and strain localisations are visible in the EBSD-KAM mappings.  Cu_6_Sn_5_-precipitates are manually segmented and divided into two classes based on the mass contrast in the FESEM-BSE micrographs and the crystallographic information obtained from the EBSD-IPF mapping in Fig. 4a and b, respectively: Cu_6_Sn_5_-precipitates situated within the recrystallised areas versus ones in the single-crystalline areas, for quantitative size comparisons. For the segmentation and class assignment, ilastik^52^ is utilised. In Fig. 4d, the segmented Cu_6_Sn_5_-precipitates are overlaid onto the FESEM-BSE micrographs from Fig. 4a. Cu_6_Sn_5_-precipitates in recrystallised areas are illustrated in pink, and those in single-crystalline areas in cyan. Again, mean precipitate radii and standard deviations are calculated. This time separately for Cu_6_Sn_5_-precipitates in recrystallised areas and ones in single-crystalline areas, printed in pink and cyan, respectively. Note, that the overall Cu_6_Sn_5_-precipitate sizes, in both recrystallised and single-crystalline areas in the SAC305-solder balls decrease with increasing Bi-content, as shown in Fig. 4e.

Figure 4d illustrates that Cu_6_Sn_5_-precipitates in recrystallised areas of all solder balls are approximately 1.5–3 times larger than in the respective single-crystalline areas, regardless of their Bi-content. Concurrently, dynamically recrystallised areas decrease with increasing Bi-content, discernible from Fig. 4b. Furthermore, a significant reduction in Cu_6_Sn_5_-precipitate coarsening in recrystallised areas from 0 to 1.1 wt% Bi is shown in Fig. 4e, whereas only a small decrease is observed between 1.1 and 1.9 wt% Bi. Conversely, Cu_6_Sn_5_-precipitate coarsening in single-crystalline areas decreases steadily with increasing Bi-content.

## Discussion

During thermo-mechanical cycling of microelectronic interconnects, a complex load case evolves within solder balls. Therein, these low-melting, low-hardness SAC-solder balls experience both prolonged time at high homologous temperatures, as well as mechanical strain due to the inherent thermal misfit stresses between the various components. In order to understand the interplay of the two main effects contributing to Cu_6_Sn_5_-precipitate coarsening during thermo-mechanical cycling, strain-induced coarsening and Ostwald ripening are studied separately and correlatively. Moreover, the impact of β-Sn matrix yield strength on strain-enhanced coarsening during thermo-mechanical cycling is evaluated by correlating three Bi-contents with Cu_6_Sn_5_-precipitate sizes and dynamic recrystallisation.

An inhomogeneous, hourglass-shaped stress distribution evolves in SAC-solder balls during thermo-mechanical cycling. This has been indicated in FEM-simulations of SAC-solder balls^39^. The hourglass-shaped stress distribution is markedly similar to the dynamically recrystallised areas in Fig. 4b. Similarly, dynamic recrystallisation occurs in the high-strain areas in the vicinity of the shear-tool contact point, whereas in lower-strain areas further away from that contact point, strain is localised in shear bands, as shown in Figs. 1 and 2. Such strain localisations are also visible in the EBSD-KAM mappings of single-crystalline areas in Fig. 4c, which may indicate where recrystallisation will occur with continued cycling^39^. Hence, strain gradients are discernible in both shear-deformed and thermo-mechanically cycled solder balls. It should be noted, however, that thermo-mechanical cycling was done for approximately 2000 cycles over the course of 2000 h and the massive shear deformation occurred in a matter of seconds, see **Methods**. The microstructural changes due to that mechanical strain, such as dynamic recrystallisation and recovery, as well as Cu_6_Sn_5_-precipitate coarsening, are considered to be primarily governed by plastic deformation. Consequently, strain-induced Cu_6_Sn_5_-precipitate coarsening is more pronounced in the recrystallised high-strain areas than in the single-crystalline lower-strain areas of thermo-mechanically fatigued solder balls, which is illustrated in Fig. 4d.

In addition to strain-induced coarsening, Cu_6_Sn_5_-precipitates also grow in the high-temperature periods during thermo-mechanical cycling due to Ostwald ripening^45^. In-situ FESEM observations of this phenomenon in Fig. 3 quantitatively confirm the overall coarsening of Cu_6_Sn_5_-precipitates, wherein bigger ones grow and smaller ones shrink^19,20,45^. However, no significant crystallographic changes such as recovery or recrystallisation occur during in-situ ageing, since both EBSD-IPF and -KAM mappings of the in-situ area before and after ageing do not exhibit any significant differences, as shown in **Supplementary Fig. 3**. Thus, plastic strain may continuously accumulate during thermo-mechanical cycling with no significant relaxation during the high-temperature periods.

Correlations between β-Sn matrix yield strength, Cu_6_Sn_5_-precipitate coarsening, recrystallisation and intergranular fatigue crack propagation are demonstrated in Fig. 4. Since Bi-additions below 2.5 wt% are in solid solution in β-Sn^53^, the main effect for the investigated Bi-contents of 1.1 and 1.9 wt% is assumed to be the increase of yield strength of the β-Sn matrix^9,10^. Despite having undergone a similar number of thermal cycles, Fig. 4e exhibits that the Cu_6_Sn_5_-precipitate sizes in the solder balls decrease with increasing Bi-content. Concurrently, the extent of dynamic recrystallisation decreases with increasing Bi-content, which is discernible in Fig. 4b. This is attributed to the strengthening effects of Bi in the β-Sn matrix, decreasing the degree of plastic deformation and dislocation generation, thus delaying dynamic recrystallisation^9,10,39^. Hence, the degree of plastic deformation in the solder ball during thermo-mechanical cycling decreases with increasing Bi-content, decelerating Cu_6_Sn_5_-precipitate coarsening and recrystallisation, thereby reducing fatigue crack propagation sites, i.e. grain boundaries^32,39^. Conversely, the increased strain-enhanced coarsening of Cu_6_Sn_5_-precipitates with decreasing Bi-content may lower their precipitate strengthening effects, which in turn may further accelerate dynamic recrystallisation and solder fatigue^8^. This is observed in Fig. 4, wherein recrystallised areas and fatigue cracking decrease with increasing Bi-content, with a concurrent decrease of overall Cu_6_Sn_5_-precipitate size. On one hand, it is discernible from Fig. 4d and e that Cu_6_Sn_5_-precipitate sizes in single-crystalline areas decrease with increasing Bi-content. This effect may be caused by Bi decreasing the overall diffusivity in β-Sn^54^, slowing diffusion-driven coarsening kinetics, which may primarily impact Ostwald ripening in lower-strain single-crystalline areas during thermo-mechanical cycling. This may lead to the steady decrease of mean precipitate radii in single-crystalline areas with increasing Bi-content observed in Fig. 4d and e, from ~ 800 nm for 0 wt%, to ~ 600 nm for 1.1 wt%, to ~ 500 nm for 1.9 wt% Bi. On the other hand, the size ratios between precipitates in recrystallised areas compared to those in single-crystalline areas decrease with increasing Bi-content in Fig. 4e. This may be attributed to the solid solution strengthening effects of Bi, primarily affecting the strain-enhanced coarsening of Cu_6_Sn_5_-precipitates in high-strain recrystallised areas. Hence, the decreased recrystallisation and the concurrent decelerated Cu_6_Sn_5_-precipitate growth with increasing Bi-content suggest a significant impact of strain-enhanced coarsening. Therein, intergranular fatigue cracks propagate through recrystallised areas, populated by coarsened Cu_6_Sn_5_-precipitates, compared to smaller ones in single-crystalline areas, see Fig. 4d. It should be noted that Fig. 4e shows a significant reduction of Cu_6_Sn_5_-precipitate coarsening from 0 to 1.1 wt% Bi and the slight additional reduction to 1.9 wt%, which follows the same trend as the fatigue crack volumes in the same alloys reported in^39^. This further highlights the correlation between Cu_6_Sn_5_-precipitate coarsening and intergranular fatigue cracking.

To summarise, Ostwald ripening of Cu_6_Sn_5_-precipitates is observed utilising in-situ FESEM ageing. However, the additional strain-enhanced coarsening of these precipitates has a more significant impact on precipitate coarsening during thermo-mechanical cycling. This is reflected in the increased precipitate sizes in dynamically recrystallised areas of thermo-mechanically cycled solder balls, compared to smaller precipitate sizes in the single-crystalline areas. Hence, Cu_6_Sn_5_-precipitate coarsening at elevated temperatures alone does not occur as fast as in combination with mechanical strain from the misfit stresses in the multi-layer device. Therefore, engineering microelectronic devices with minimal thermal misfit stresses may delay Cu_6_Sn_5_-precipitate coarsening in SAC-solder ball interconnects. Decelerated precipitate coarsening may in turn have a positive effect on the fatigue life of solder balls, as precipitate-strengthening effects on yield strength are retained for a larger amount of thermo-mechanical cycles. This is illustrated by the fact that the overall Cu_6_Sn_5_-precipitate size decreases with increasing Bi-content, i.e. increasing matrix yield strength. Not only do Bi-additions to SAC305 delay dynamic recrystallisation, but also Cu_6_Sn_5_-precipitate coarsening, which may maintain their homogeneous distribution and strengthening effect over a larger amount of thermal cycles. These mechanistic insights into precipitate coarsening may help in the development of materials science-based device designs to prolong the lifetime of microelectronic interconnects.

## Methods

### Materials and sample fabrication

The investigated solder balls are produced by droplet spraying in an inert N_2_ atmosphere and subsequently soldered between the Cu metallisation of the chip and the PCB. Reflow is done at a peak temperature of 240 °C and with a mean heating rate of 44 °C/min in inert N_2_ atmosphere, followed by rapid air cooling to 90 °C with a mean cooling rate of 107 °C/min and ambient air cooling to room temperature. The as-reflowed solder balls which are shear-deformed and in-situ aged, are Sn – 3.0 wt% Ag – 0.5 wt% Cu (SAC305). The thermo-mechanically cycled samples SAC305, SAC305 + 1.1 wt% Bi and SAC305 + 1.9 wt% Bi are cycled 1764, 2914 and 2570 times, respectively. Thermo-mechanical cycling is conducted between − 40 and 125 °C with ramp- and dwell-times of 15 min, respectively by alternately injecting hot and cold air into a furnace. A total of twelve solder balls were analysed in this study, including four additional thermo-mechanically cycled samples added during the review process. Those consist of: one as-reflowed SAC305 solder ball, four shear-deformed solder balls, one shear-deformed and in-situ aged solder ball, and six thermo-mechanically cycled solder balls—two each of SAC305, SAC305 + 1.1 wt% Bi, and SAC305 + 1.9 wt% Bi.

## Shear deformation experiments

Plastic shear deformation of as-reflowed solder balls is performed with a xyzTec Sigma Condor bond-tester. A variety of maximum shear forces (F_max_) are set as stop criteria for the experiments in order to achieve different degrees of deformation: 3 N, 5 N, when the force drops 0.5 N from F_max_ and when it drops 1 N from F_max_. The schematic experimental setup is shown in **Supplementary Fig. 1a** and the force – time curves are given in **Supplementary Fig. 1b** for the investigated stop criteria.

### Sample preparation for FESEM

All cross-sections are pre-prepared with a 3D Micromac microPREP PRO femtosecond laser with a laser power of 300 mW. For in-situ FESEM ageing, the backside of the sample is also cross-sectioned, in order to ensure thermal contact with the MEMS heating chip. The final cross-section for FESEM imaging and EBSD mapping are prepared with a Hitachi IM4000 + ion milling system. The accelerating voltage for ion milling is set to 6 kV and the swing angle to 30°.

## Ex-situ FESEM imaging

All ex-situ FESEM micrographs and EBSD mappings are acquired at room temperature with a ZEISS GeminiSEM 450 FESEM. For FESEM-BSE imaging, an acceleration voltage of 3 kV is utilised. EBSD mappings are done with an accelerating voltage of 10 kV, a step size of 400 nm and an Oxford Symmetry detector. EDX mapping is done with an acceleration voltage of 3 kV and an Oxford Ultim Extreme detector. Oxford Instrument AZtecCrystal 6.1 is used for the evaluation of EBSD and EDX data.

## In-situ FESEM ageing

In-situ ageing is done in a ZEISS Sigma 560 VP FESEM, utilising a Norcada SEM in-situ heating and biasing holder (NHB-Z-SNL-001), equipped with Norcada MEMS heating chip (ZHTN-1000 L, 1.6 mm dia. heater). The ageing temperature is set according to the time – temperature treatment shown in **Supplementary Fig. 2** and FESEM-BSE micrographs are acquired at the timesteps T1 – T4, corresponding to T1 = 0 min/125°C, T2 = 20 min/125°C, T3 = 20 min/125°C + 30 min/175°C and T4 = 20 min/125°C + 90 min/175°C.

### Cu_6_Sn_5_-precipitate segmentation, calculation of equivalent radii and in-situ ageing heatmap

The segmentation of Cu_6_Sn_5_-precipitates in as-reflowed and shear-deformed solder balls is done with binary thresholding of FESEM-BSE micrographs. For the segmentation of Cu_6_Sn_5_-precipitates in thermo-mechanically cycled solder balls, ilastik 7.1.0^52^ is utilised. In both cases, their respective areas and hence their equivalent radii are then calculated from these thresholded images, utilising Python 3.8.13, OpenCV 4.0.1 and numpy 1.22.3 and matplotlib 3.5.1. Additional information is provided in **Supplementary Notes 1** and **3**. The heatmap depicting Cu_6_Sn_5_-precipitate morphology changes during in-situ FESEM ageing in Fig. 3d is generated by subtracting the binarily thresholded images of the analysis area from one another. Matplotlib 3.5.1 is utilised for the visualisation.

## Supplementary Information

Below is the link to the electronic supplementary material.

Supplementary Material 1

## Data Availability

The data that support the findings of this study are available from the corresponding author upon reasonable request.

## Code availability

All code that support the findings of this study are available from the corresponding author upon reasonable request.
