# Supplementary material for: Unravelling Cu6Sn5 precipitate coarsening mechanisms in SAC solders under thermomechanical cycling
Source: Sci Rep. 2025 Oct 28;15:37642. doi: 10.1038/s41598-025-21633-y (PMC12569173; doi:10.1038/s41598-025-21633-y)
Supplement: Supplementary file 1 — Supplementary Material 1 [file 41598_2025_21633_MOESM1_ESM.pdf]

Unravelling Cu_6Sn_5 precipitate coarsening mechanisms in SAC solders under thermomechanical cycling

Charlotte Cui[1], Sebastian Krauß[2], Hooman Hosseinkhannazer[3], Julien Magnien[1], Olena Vertsanova[2], Michael Reisinger[4], Peter Imrich[4], Walter Hartner[5], Roland Brunner[1*]

[1] Materials Center Leoben Forschung GmbH, Department Microelectronics, Vordernbergerstraße 12, 8700 Leoben, Austria

[2] Carl Zeiss AG, Carl-Zeiss-Straße 22, 73447 Oberkochen, Germany

[3] Norcada Inc, 4548 99 Street NW, Edmonton, AB T6E 5H5, Canada

[4] Kompetenzzentrum für Automobil- und Industrieelektronik GmbH, Europastraße 8, 9524 Villach, Austria

[5] Infineon Technologies AG, Wernerwerkstraße 2, 93049 Regensburg, Germany

*Corresponding author: roland.brunner@mcl.at

This study presents the effects of mechanical strain and ageing, as well as their interplay during thermo-mechanical cycling, on Cu_6Sn_5-precipitate coarsening in SAC-solder balls in microelectronic devices. Ageing is performed utilising in-situ FESEM imaging, whereas plastic deformation and thermo-mechanical cycling are performed ex-situ. Supplementary information is provided in the following.

Supplementary Figure 1: Experimental setup for shear deformation and force – time-curves.

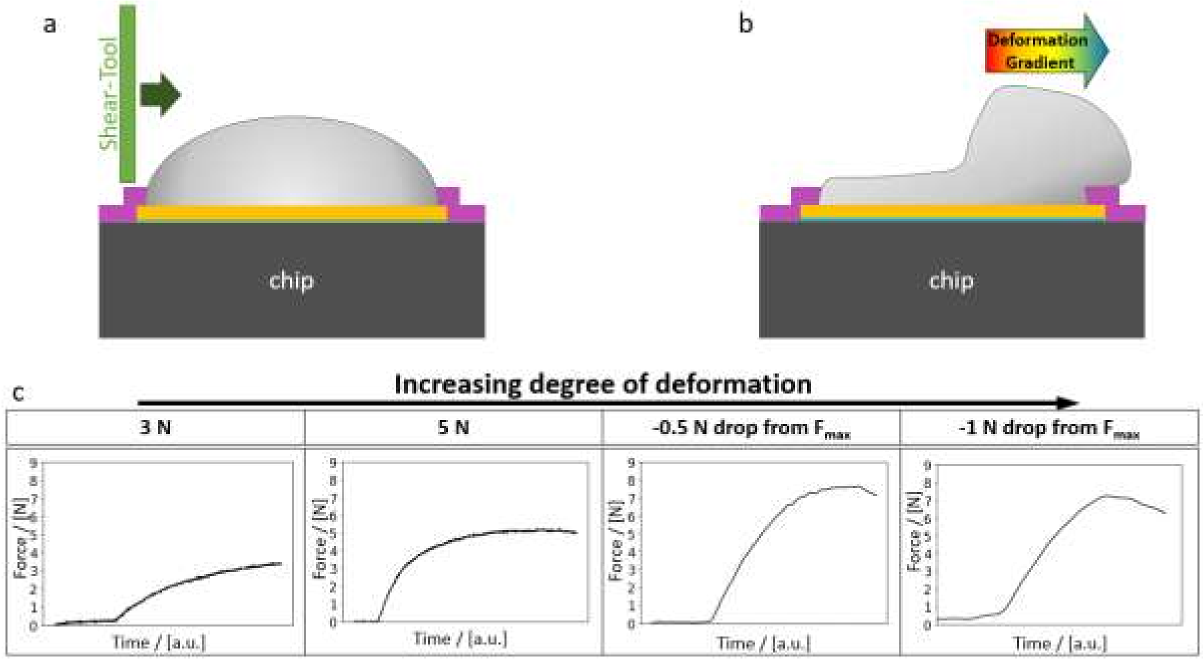

a Schematic solder ball before shear deformation and sketched shear-tool, depicted in green, and shear direction, illustrated as green arrow. The solder ball is illustrated in bright grey, whereas the chip is shown in dark grey. **b** Schematic solder ball after shear deformation. The deformation gradient within the ball is depicted with an arrow. **c** Force – time-curves for the shear experiments of the solder balls shown in **Fig. 1**. From left to right: increasing degree of solder ball deformation. Force is scaled from 0 to 9 N in all curves. Time is scaled arbitrarily, since the deformation experiments are of various durations.

Supplementary Note 1: Threshold-based segmentation of Cu_6Sn_5-precipitates.

In order to evaluate Cu_6Sn_5-precipitate sizes in the as-reflowed and shear deformed conditions, as well as during in-situ FESEM ageing, they are binarily segmented from the respective FESEM-BSE micrographs. For that, grey value thresholds are set according to the mass contrast of Cu_6Sn_5-precipitates in those micrographs, their area is evaluated and from that, their equivalent radii are calculated. Python 3.8.13, OpenCV 4.0.1 and numpy 1.22.3 and matplotlib 3.5.1 are utilised for the evaluation and visualisation.

Supplementary Note 2: In-situ FESEM ageing.

For the study of Cu_6Sn_5-precipitate coarsening during the high-temperature periods of thermo-mechanical cycling, in-situ FESEM ageing is performed. The in-situ ageing experiment is conducted in a Zeiss Sigma FESEM utilising a Norcada MEMS heating chip and the temperature profile shown in **Supplementary Fig. 2**. In order to ensure thermal contact to the MEMS heating chip, the sample backside is flattened utilising a 3D Micromac microPREP PRO femtosecond laser with a laser power of 300 mW. In-situ FESEM-BSE imaging is done with 10 kV.

Supplementary Figure 2: In-situ FESEM ageing treatment.

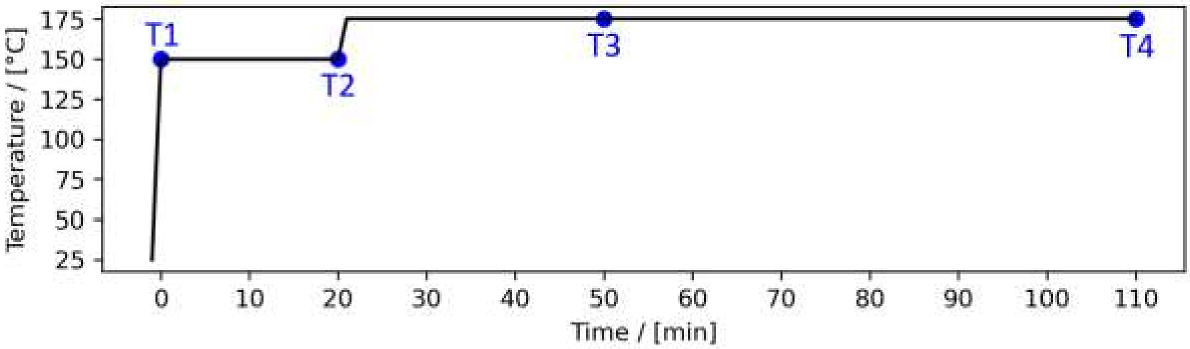

Schematic time – temperature diagram of the in-situ FESEM ageing treatment. T1 – T4 mark the timesteps where FESEM-BSE micrographs in **Fig. 3** are acquired and analysed.

Supplementary Figure 3: EBSD mappings of the in-situ area before and after ageing treatment.

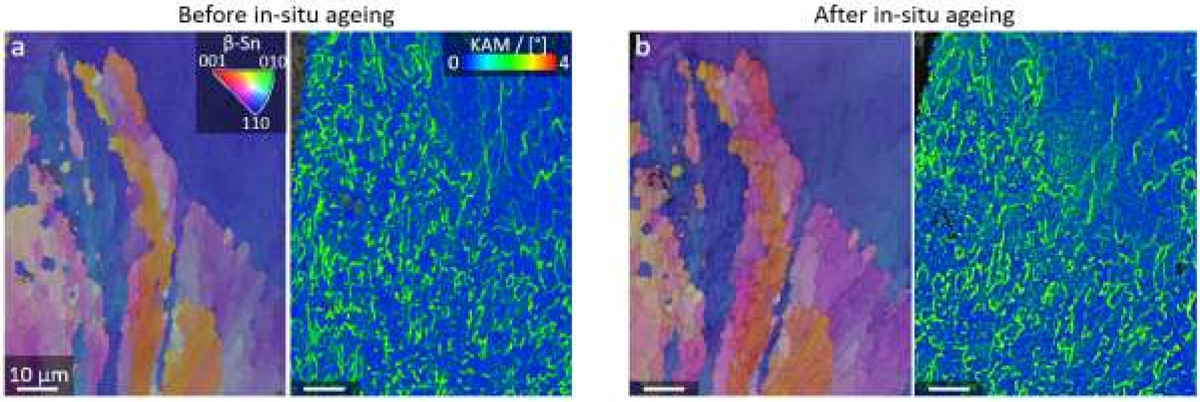

EBSD-IPF and -KAM mappings of the in-situ area **a** before ageing and **b** after ageing. Scalebar of 10 µm is valid for all images. KAM is ranged from 0 to 4 °, respectively.

Supplementary Note 3: Segmentation of Cu_6Sn_5-precipitates in single crystal and recrystallised areas.

For the distinction of Cu_6Sn_5-precipitates in recrystallised and single crystal areas of thermo-mechanically fatigued solder balls, the precipitates are labelled separately utilising ilastik 7.1.0. Those labels are then evaluated utilising Python 3.8.13, OpenCV 4.0.1 and numpy 1.22.3 and matplotlib 3.5.1. Similar to the evaluation of threshold-based segmentation in **Supplementary Note 1**, the areas and equivalent radii are evaluated based on the labelled image data.
